# Editorial: Multisystem inflammatory syndrome in children

**DOI:** 10.3389/fped.2024.1370467

**Published:** 2024-03-19

**Authors:** Angela Mauro, Teresa Giani

**Affiliations:** ^1^ Rheumatology Unit, Department of Pediatrics, ASST Fatebenefratelli-Sacco, Milan, Italy; ^2^ Department of Pediatrics, Meyer Children’s University Hospital, Florence, Italy

**Keywords:** multisystem inflammatory syndrome in children (MIS-C), PIMS-TS, Kawasaki disease (KD), COVID-19, children

**Editorial on the Research Topic** Multisystem inflammatory syndrome in children

##  

Multisystem inflammatory syndrome is a severe complication associated with COVID-19, initially recognized as a distinct clinical entity in 2020 (1). In a bulletin from the UK's National Health Service (NHS) in late April 2020, highlighting a new multi-system inflammatory condition involving a small number of children, the term “PIMS” (Pediatric Inflammatory Multisystem Syndrome associated with SARS-CoV-2) was initially used. Primarily observed in pediatric patients, it was subsequently classified by the Centers for Disease Control and Prevention as Multisystem Inflammatory Syndrome in Children (MIS-C) (2). MIS-C manifests as a rare delayed hyperinflammatory response following SARS-CoV-2 infection. Although the exact pathophysiology remains unclear, the SARS-CoV-2 coronavirus seems to trigger a dysregulated pathological immune response in the host, leading to systemic vasculitis and widespread acute organ damage (3).

Like the viral infection caused by COVID-19, also other infectious agents, such as the Epstein-Barr virus (EBV), have also provided the opportunity to analyze the complex mechanisms leading to hyperinflammatory states. EBV can influence the expression and modulation of TLR7 and TLR9 signaling pathways, and consequently the transcription factor NF-κB activation (Tan et al.) (4). Liu et al. observed how the type of interplay between EBV and TLRs defines the disease outcome. Patients with chronic active EBV (CAEBV) showed a sustained and heightened activation of TLR7 and TLR9 along with their downstream signaling mediators (Liu et al.). This could suggest how a deficit in the self-regulation of negative feedback mechanisms associated with TLRs predisposes to a prolonged, and excessive inflammatory response, potentially contributing to an unfavorable outcome.

MIS-C presents with a heterogeneous clinical profile and variable severity, involving multiple organs and characterized by a state of hyperinflammation, often requiring intensive care. It shares clinical similarities with Kawasaki Disease (KD)-like shock syndrome, explaining how treatment protocols have been mostly derived from those used in KD (5).

About cardiac manifestations of these diseases, myocarditis and left ventricular systolic dysfunction are very common in MIS-C patients and rare in KD. Regarding the occurrence coronary aneurysm (CAA), a typical complication of KD, after the introduction of intravenous immunoglobulin treatment, they can be seen only in 4% of the patients, while in MIS-C, 14%–36% exhibit CAA (6).

Difference between MIS-C and KD has shown in Table 1.

**Table 1:** Difference between multisystem inflammatory syndrome in children and Kawasaki disease.

	MIS-C	KD
Age	8–11 years	Infants and children <5 years of age
Sex ratio (M/F)	1:1	1.5:1
Gastrointestinal symtoms	+	−
Myocardial dysfunction	+	−
Coronary Aneurism	+	−
Organ dysfunction	+	−
Inflammatory markers	+	+
Fatality rate	1.4%–1.7%	0.01%

This new condition has attracted significant scientific interest and has resulted in numerous publications (7–12), (Table 2). However, despite advancements, many aspects of MIS-C, including epidemiology, pathogenesis, clinical spectrum, and long-term outcomes, still remain poorly understood, providing numerous avenues for future research. Furthermore, although it has become increasingly clear that MIS-C and KD exhibit significant differences, some intriguing points of intersection seem to exist in their pathogenetic mechanisms that still need to be defined (13).

**Table 2:** Top 6 articles related to multisystem inflammatory syndrome, MIS-C, updated as of January 29, 2019.

Year	Title	First author	Journal	References	NIH percentile	RCR
2021	American college of rheumatology clinical guidance for multisystem inflammatory syndrome in children associated with SARS-CoV-2 and hyperinflammation in pediatric COVID-19: version 3	Henderson LA	Arthritis Rheumatol.	(7)	99.8	31.97
2020	Clinical characteristics of children and young people admitted to hospital with COVID-19 in united kingdom: prospective multicentre observational cohort study	Swann OV	BMJ	(8)	99.7	29.18
2020	Multisystem inflammatory syndrome in children associated with severe acute respiratory syndrome coronavirus 2 infection (mis-c): a multi-institutional study from New York City	Kaushik S	J Pediatr.	(9)	99.6	23.26
2020	American college of rheumatology clinical guidance for multisystem inflammatory syndrome in children associated with SARS-CoV-2 and hyperinflammation in pediatric COVID-19: version 1	Henderson LA	Arthritis Rheumatol.	(10)	99.6	22.40
2021	Association of intravenous immunoglobulins plus methylprednisolone vs. immunoglobulins alone with course of fever in multisystem inflammatory syndrome in children	Ouldali N	JAMA	(11)	99.5	21.13
2020	Multisystem inflammatory syndrome in children and COVID-19 are distinct presentations of SARS-CoV-2	Diorio C	J Clin Invest.	(12)	99.4	19.09

RCR, relative citation ratio.

At the beginning of the pandemic, differentiating children with acute severe COVID-19 infection from those who developed the post-infectious hyperinflammatory syndrome, MIS-C, proved challenging (14). In this regard, Jiju et al. in their retrospective study compared the features of 161 symptomatic acute COVID-19 and 50 MIS-C patients (≤19 years) admitted to a tertiary pediatric hospital in the North-West of England (Jiju et al.). They observed that MIS-C patients were older, with a median of 10.3 years, developed the disease later with respect to the primary infection, and often had associated comorbidities. Clinically, they typically presented with abdominal and neurological symptoms, higher inflammatory markers, and showed a more severe disease course, with a higher incidence of death.

European countries and the United States mostly contributed to the majority of scientific publications regarding MIS-C at the onset of the pandemic (15, 16). Conversely, MIS-C has rarely been reported in Chinese children. The reasons could be due to differences in prevalence rates of infection in children and differences in ethnic, genetic background, and SARS-CoV-2 subtypes (17). Wang et al. described a 4-year-old Chinese girl with severe COVID-19 infection complicated with MIS-C successfully treated according to an expert consensus statement Wang et al. (18).

MIS-C may present with a variety of clinical presentations, also in terms of severity. Efforts have been made to comprehend the role of biomarkers in predicting the disease course and outcome (19, 20). For example, older age and initial serum albumin levels have been identified as early indicators, helping to recognize children at high risk for intensive care unit admission (21).

Snipaitiene et al. in a retrospective study involving 43 patients evaluated the role of platelet (PLT) count and PLT indices (plateletcrit, mean platelet volume, and platelet distribution width) in predicting MIS-C severity in children who presented at the Hospital of Lithuanian University of Health Sciences Kauno Klinikos. They found that these markers allowed better prediction of MIS-C severity (Snipaitiene et al.). With the same purpose, Fastiggi et al. analyzed the role of the thyroid axis, Euthyroid Sick Syndrome (ESS), in a single-center observational study involving 42 patients with MIS-C, showing it to be a potential predictor of severe MIS-C course (Fastiggi et al.).

The first-line treatment for MIS-C, usually based on intravenous immunoglobulin (IVIG) and corticosteroids, aim to address the inflammatory response and symptoms associated with this condition (22).

In some cases, additional therapies may be considered based on the severity of the condition and individual patient needs. Among the treatments included in the expert consensus statement anakinra has been extensively used in this condition (23). However, data on the efficacy and safety of anakinra in patients with MIS-C are still lacking (16). Licciardi et al. in their retrospective multicenter study compared patients treated with anakinra in the ICU with those treated in the pediatric wards, observing that anakinra resulted to be efficacious and safe (Licciardi et al.).

In a MIS-C patients resistant to first line therapy, Tocilizumab, anti-IL 6, has been studied as a second line of treatment. In fact Çelikel et al. investigated the efficacy of anakinra, and/or tocilizumab in resistant patients with severe MIS-C admitted in the PICU. They enrolled at 33 patients with MIS-C with a median age of 9 years. All the patients were given first line of therapy. 23/33 (69.9) patients took Anakinra. Two patients were switched to tocilizumab because they were unresponsive to anakinra. All patients showed an increase in lymphocyte and platelet counts and a decrease in ferritin, B-type natriuretic peptide, and troponin levels after first week of treatment (24).

Moreover, Niño-Taravilla et al. describe a case of 8-year-old boy with severe MIS-C treated with tocilizumab (8 mg/kg) and corticosteroid therapy. Two days after the start of treatment, he showed an improvement in symptoms, cardiac function and laboratory tests (25).

Intravenous immunoglobulins (IVIG) is the well-established primary therapeutic option for KD and has also been extensively used in MIS-C (18). However, IVIG may entail rare, often under-recognized side effects such as headaches, hyperviscosity, and hemolysis. The passive transfer of isoagglutinins, typically anti-A or anti-B antibodies, has been recognized as the main causative factor for IVIG-associated hemolytic anemia (26).

In the context of KD, IVIG-associated hemolysis affects up to 16% of the patients. Sedlin et al. reported the first two pediatric patients, a 2 and an 8-year-old girl, diagnosed with MIS-C developing this adverse effect after IVIG therapy (27).

Evidence regarding second-line therapy in patients with resistant KD remains contradictory, a second infusion of IVIG still represents one of the most frequently employed options in such cases (Sedlin et al.). However, given the side effects associated with high IVIG doses and the availability of alternative treatments, several studies have explored their efficacy and side effects (28). Pan et al. conducted a network meta-analysis and used an aggregate Data Drug Information System software v.1.16.5 incorporating clinical trials comparing the safety and efficacy of infliximab, second IVIG infusions, and intravenous pulse methylprednisolone (IVMP) Pan et al. (29). Infliximab emerged as the optimal second-line treatment choice, although associated with an increased susceptibility to hepatomegaly. No significant differences in the risk of developing a coronary artery aneurysm among these three different options emerged.

KD and MIS-C are now recognized under the same hyperinflammatory umbrella, yet they are considered distinct diseases. However, during the early stages of the pandemic, the emergence of a new condition with a KD-like presentation posed diagnostic challenges. Initially, the boundaries between MIS-C and KD seemed blurred, sparking renewed interest among clinicians and scientists in the study of KD (30). Tan and colleagues in their bibliometric analysis have explored the current hotspots and trends in research, founding that in the period 2017–2021, more than 5,500 articles on KD have been published in the Web of Science and Scopus databases, predominantly authored by researchers from Japan, the USA, and China, with a specific focus on “COVID-19” and “multisystem inflammatory disease” (Pan et al.).

In conclusion, despite the profound impact of the COVID-19 pandemic, it has nonetheless left a significant mark on the scientific landscape. This is evident in the collaborative research endeavors that undeniably contributed to advancements in medical knowledge.
